# A Screening of the MMV Pandemic Response Box Reveals Epetraborole as A New Potent Inhibitor against *Mycobacterium abscessus*

**DOI:** 10.3390/ijms22115936

**Published:** 2021-05-31

**Authors:** Taeho Kim, Bui-Thi-Bich Hanh, Boeun Heo, Nguyenthanh Quang, Yujin Park, Jihyeon Shin, Seunghyeon Jeon, June-Woo Park, Kirandeep Samby, Jichan Jang

**Affiliations:** 1 Division of Applied Life Science (BK21 Four Program), Research Institute of Life Science, Gyeongsang National University, Jinju 52828, Korea; taeho12349@gmail.com (T.K.); hanhm0515006@gstudent.ctu.edu.vn (B.-T.-B.H.); 2 Molecular Mechanisms of Antibiotics, Division of Life Science, Department of Bio & Medical Big Data (BK21 Four Program), Research Institute of Life Science, Gyeongsang National University, Jinju 52828, Korea; hbo0113@naver.com (B.H.); nguyenthanhquang1411@gmail.com (N.Q.); syryejr@naver.com (Y.P.); jihyeon2531@naver.com (J.S.); 3 Division of Life Science, Gyeongsang National University, Jinju 52828, Korea; sarahpop@naver.com; 4 Department of Environmental Toxicology and Chemistry, Korea Institute of Toxicology, Jinju 52843, Korea; jwpark@kitox.re.kr; 5 Human and Environmental Toxicology Program, Korea University of Science and Technology (UST), Daejeon 34113, Korea; 6 Medicines for Malaria Venture (MMV), 20, Route de Pré-Bois, 1215 Geneva, Switzerland; sambyk-consultants@mmv.org

**Keywords:** *Mycobacterium abscessus*, epetraborole, benzoxaboroles, drug discovery, antibiotics

## Abstract

*Mycobacterium abscessus* is the one of the most feared bacterial respiratory pathogens in the world. Unfortunately, there are many problems with the current *M. abscessus* therapies available. These problems include misdiagnoses, high drug resistance, poor long-term treatment outcomes, and high costs. Until now, there have only been a few new compounds or drug formulations which are active against *M. abscessus,* and these are present in preclinical and clinical development only. With that in mind, new and more powerful anti-*M. abscessus* medicines need to be discovered and developed. In this study, we conducted an in vitro-dual screen against *M. abscessus* rough (R) and smooth (S) variants using a Pandemic Response Box and identified epetraborole as a new effective candidate for *M. abscessus* therapy. For further validation, epetraborole showed significant activity against the growth of the *M. abscessus* wild-type strain, three subspecies, drug-resistant strains and clinical isolates in vitro, while also inhibiting the growth of *M. abscessus* that reside in macrophages without cytotoxicity. Furthermore, the in vivo efficacy of epetraborole in the zebrafish infection model was greater than that of tigecycline. Thus, we concluded that epetraborole is a potential anti-*M. abscessus* candidate in the *M. abscessus* drug search.

## 1. Introduction

Despite great strides in the field of infectious disease over the past century, *Mycobacterium abscessus* (hereafter referred to as *Mab*) complex infection has remained a leading cause of morbidity and mortality in cystic fibrosis (CF) patients [1]. *Mab* infections are deadly complicated to treat due to their mechanisms for drug resistance and biofilm generation [2]. Although there is no compelling agent for *Mab* treatment: clarithromycin (CLA), amikacin (AMK), and cefoxitin (CFX) are currently used as a drug combination for 1 to 2 months, followed by an oral maintenance regimen, usually with a fluoroquinolone, based on the recommendations of experts [3]. However, long-term and intensive combination therapy has shown high rates of treatment failure, recurrences, and adverse effects. Furthermore, the use of this combination therapy resulted in only a 50% efficacy rate even with adjunctive surgery and most patients either relapse or die [4]. Therefore, more efficacious drugs are urgently needed.

In this context, high throughput screening has been conducted with *Mab* using various chemical libraries [5,6,7,8,9,10,11,12]. Unfortunately, there are not many active new drug candidates in either the clinical or discovery phase. This may be due to the extremely low hit rate of chemical drug screens that target *Mab* [6]. Even selected hits have not been further developed successfully into clinical trials. Fundamentally, mycobacteria contain a very thick cell membrane called mycomembrane which is composed of peptidoglycan, arabinogalactan covalently linked to an inner leaflet of long-chain mycolic acids. Furthermore, *Mab* also contains a large variety of extractible lipids from the outer leaflet of the mycomembrane, such as glycopeptidolipids (GPLs) [13,14]. Thus, these thick cell envelopes are a major permeability barrier for drug penetration. Besides, *Mab* is an intracellular pathogen. During infection, *Mab* is engulfed by human macrophages, overcomes a host’s immune defense mechanisms, replicates, and resides inside human immune cells [15]. Therefore, only compounds with a high enough penetration ability to pierce the macrophage plasma membrane and the thick cell membrane of the *Mab* will lead to an inhibition of intracellular mycobacterial growth. To make matters worse, *Mab* also contains efflux pump-related genes that can export chemical agents, as well as possessing some enzymes that can modify drugs and targets which makes it difficult for drugs to be functional [16,17,18].

Here, we screened the Pandemic Response Box against *Mab* to identify a promising anti-*Mab* agent. The Pandemic Response Box is a drug library assembled from the Medicines for Malaria Venture (MMV) and Drugs for Neglected Diseases initiative (DNDi), in association with scientists from both industry and academia, to foster new research into treatments for pandemic diseases. This library contains 400 structurally diverse compounds (201 antibacterials, 153 antivirals, and 46 antifungals) for screening against infective and neglected diseases. Using this valuable library, we identified one potent candidate named epetraborole (also known as AN 3365; code number: GSK 2251052) that showed excellent growth inhibitory activity against *Mab* in the low nanomolar range. We further characterized the hit molecules regarding their bacterial inhibitory effect inside both macrophage and zebrafish models of infection.

## 2. Results

### 2.1. Drug Screening against Mab Using a Pandemic Response Box

**Figure 1:**
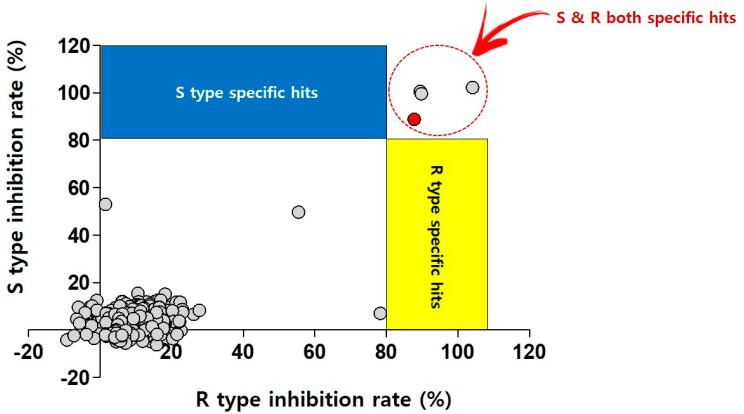
Dual screening of the Pandemic Response Box using *Mab* S and R variants. Scatter plot distribution showing the results of the *Mab* S and R variant dual screening of the Pandemic Response Box using resazurin reduction assay. A total of 400 compounds from the Pandemic Response Box were screened at 20 μM against both *Mab* S and R variants. Growth inhibition of at least 80% was defined as the cut-off which resulted in 3 hits (0.75% hit rate). Red closed circle indicates CLA as positive control.

For the screen, the library was obtained in plates from the Medicines for Malaria Venture (MMV, Geneva, Switzerland). It was then diluted to 2 mM and transferred directly into assay plates to yield a primary screen concentration of 20 μM (final concentration of 2% DMSO). The primary screen conditions were used for two different strains (*Mab* subsp. *abscessus* CIP 104536T S- and R-variants) and 80% growth inhibition was plotted. The single-point assays produced 3 compounds (primary hit rate 0.75%) that had a >80% inhibitory effect against both *Mab* variants in cation-adjusted Mueller–Hinton (CAMH) medium (Figure 1).

The three hits were epetraborole (ETB), eravacycline (ERV), and bedaquiline (BDQ). The 3 active hits were then confirmed by dose-response curve determination to generate inhibitory concentration (IC) by resazurin microtiter assay (REMA) and their half inhibitory concentrations (IC_50_) were below 1 µM, which is more potent to that of reference compound CLA. The IC_50_ values were 0.4, 0.1 and 0.8 μM for ETB, ERV, and BDQ, respectively (Table 1). Because recent studies have shown the anti-*Mab* effect of ERV and BDQ, we will describe the findings of only ETB against *Mab* in this study.

### 2.2. ETB Has Mab Killing Effect and Exhibits Potent Activity against Mab Subspecies, Clinical Isolates, and Drug-Resistant Strains

**Figure 2:**
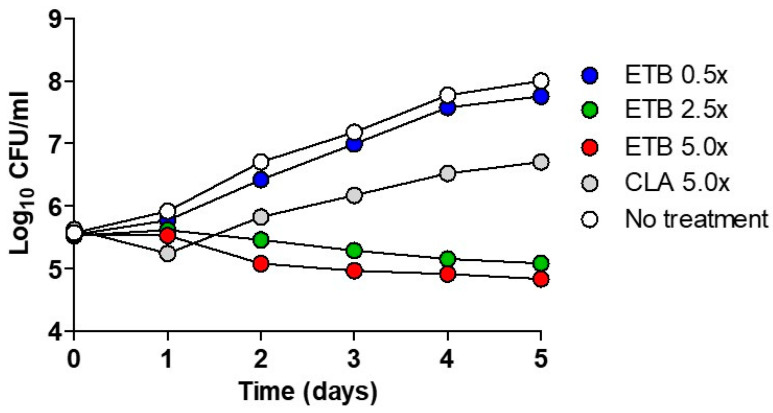
Time–kill curves of ETB against *Mab*. The bacteria were grown in a liquid culture (Middlebrook 7H9 medium) in the presence of the indicated concentrations of ETB and was plated on a 7H10 Middlebrook agar plate. Antibiotic concentrations are indicated by different symbols. Each point represents the mean of triplicate determinations.

Time-kill assays were performed using ETB for the *Mab*-S variant (CIP 104536^T^). Figure 2 shows the pattern of growth and killing of *Mab* by ETB at different concentrations. The CLA was used as a negative control. After a short declined lag phase at day 1, an increase in CFU (colony forming unit) was observed at 5× CLA concentration. Furthermore, it reached the highest CFU counts after 5 days of incubation. In contrast, ETB exhibited bactericidal activity at concentrations of 2.5-, and was 5-fold higher than the IC_50_ determined by the REMA (Table 1). Only 1 day later, ETB started to decrease the bacterial density with its maximum decrease after 5 days at 2.5× and 5× concentrations of IC_50_. The bacterial killing effect was not observed at 0.5× MIC of ETB.

We continued to evaluate the concentrations that caused inhibition of growth of *Mab* using ETB for three different *Mab* subspecies comprising *Mab* subsp. *abscessus* CIP 104536^T^*, Mab* subsp. *massiliense* CIP108297^T^, and *Mab* subsp. *bolletii* CIP108541^T^. As shown in Table 2, a decrease in fluorescence was observed, indicating a dose-dependent inhibitory effect after incubating *Mab* strains with the ETB for 5 days. All the subspecies tested were susceptible to ETB. *Mab* subsp. *massiliense* CIP108297^T^ showed the lowest IC value (IC_50_ values of 0.11 µM and MIC_90_ values of 0.33 µM) and *Mab* subsp. *abscessus* CIP 104536^T^ showed the highest IC_50_ and IC_90_ value at 0.25 µM and 0.56 µM respectively. Meanwhile, *Mab* subsp. *bolletii* CIP108541^T^ was 0.22 µM for IC_50_.

In addition, we conducted IC determination tests to know whether ETB could inhibit the growth of drug-resistant strains that were laboratory induced at high concentrations of amikacin (AMK), cefoxitin (CFX), and CLA as previously described [19]. As shown in Table 2, all the AMK, CFX, and CLA resistant mutants were sensitive to ETB. ETB showed very close IC values when compared to wild-type *Mab* subsp. *abscessus* CIP104536^T^, to all AMK, CFX, and CLA resistant mutants. These results demonstrate that ETB is an active compound against wilt-type *Mab*, as well as against AMK, CFX, and CLA resistant strains. Thus, ETB may be used for treatment in AMK, CFX, and CLA sensitive and resistant *Mab* infected patients in clinics. To understand ETB in vitro activity better, we also tested ETB in vitro susceptibility test against clinical isolates. The clinical isolate set was organized with S and R variants in *Mab* subsp. *abscessus* and *Mab* subsp. *massiliense*. As shown in Table 2, the growth of *Mab* clinical isolates was significantly decreased in ETB dose-dependent manner. For example, the growth of *Mab* subsp. *abscessus* KMRC 00136-61039 S variants and *Mab* subsp. *massiliense* KMRC 00200-61202 R variants was inhibited at IC_50_ values of 0.29 µM and 2.75 µM of ETB respectively. Most clinical isolates showed growth inhibition at the much lower IC_50_ range of 0.29–2.75 µM. Taken altogether, these results indicate that ETB can be considered as an effective drug candidate to cure drug-sensitive and resistant *Mab* infections regardless of variants in clinic.

### 2.3. Epetraborole Is Non-Toxic to Macrophage and Inhibits Intracellular Growth of Mab

**Figure 3:**
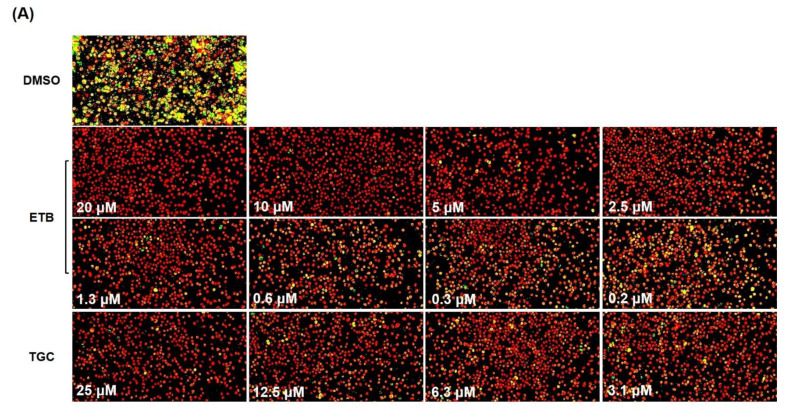

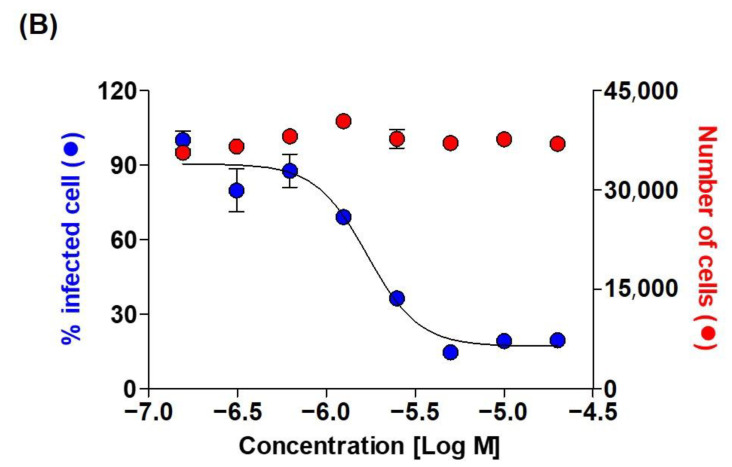
Intracellular activity of ETB against *Mab*S-mWasabi. (**A**) Images of *Mab*S-mWasabi infected mBMDMs on day 3 after treatment with different doses of ETB and TGC. DMSO was used as negative control. After 3 days of incubation with antibiotics, mBMDMs were stained with syto60 (red), and the cells were analyzed using the automated cell imaging system. The yellow colors represent *Mab*S-mWasabi that were phagocytized by red-stained mBMDM cells. The pixel intensities of live *Mab*S-mWasabi (blue closed circle) and cell number (red closed circle) were quantified after treatment with ETB (**B**) using CellReporterXpress^®^ Image Acquisition and Analysis Software.

In order to evaluate the activity of ETB against *Mab* that reside in macrophages, we tested its potency using a macrophage-based phenotypic assay with an automated cell imaging system. To confirm the colonization by *Mab* inside a macrophage under treatment with different concentrations of ETB, we used a *Mab* strain carrying a mWasabi gene which encoded monomeric green fluorescent protein. Using this system, we monitored the intracellular growth of mWasabi protein-expressing *Mab* subsp. *abscessus* CIP104536^T^ S variant (hereafter referred to as *Mab*S-mWasabi) in bone marrow-derived macrophages (mBMDM). mBMDM cells were seeded at 7 × 10^5^/well and infected with *Mab*S-mWasabi that were mixed at a multiplicity of infection (MOI) of 1:1. As shown in Figure 3A, the strong intracellular mWasabi pixel intensity was quantified in DMSO-treated cells (Figure 3A). However, ETB showed significant activity against *Mab* S-mWasabi replication in mBMDM in a concentration-dependent manner. The positive control tigecycline (TGC) has also demonstrated good intracellular activity against *Mab*S-mWasabi in a dose-dependent manner. The percentage of pixel intensity of intracellular *Mab*-mWasabi and number of live cells at different concentrations of ETB were quantified. The mBMDM that harbors *Mab*S-mWasabi treated with various concentrations (20, 10, 5, 2.5, 1.3, 0.6, 0.3, and 0.2 μM) of ETB showed significantly reduced mWasabi pixel intensity (Figure 3B). This result demonstrates that ETB has an ability to inhibit bacterial growth inside the cell.

### 2.4. ETB Is also Excellent Antimicrobial Agent for Mab in Danio Rerio

**Figure 4:**
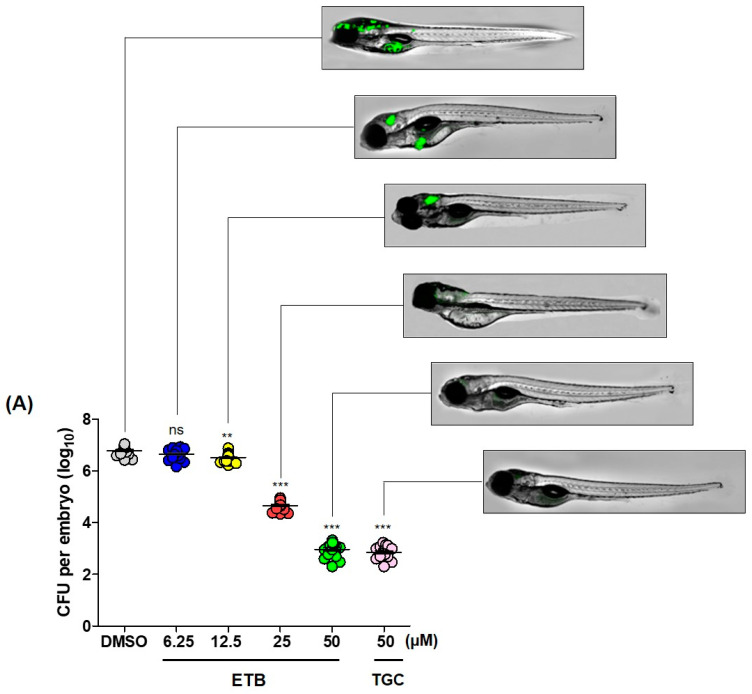

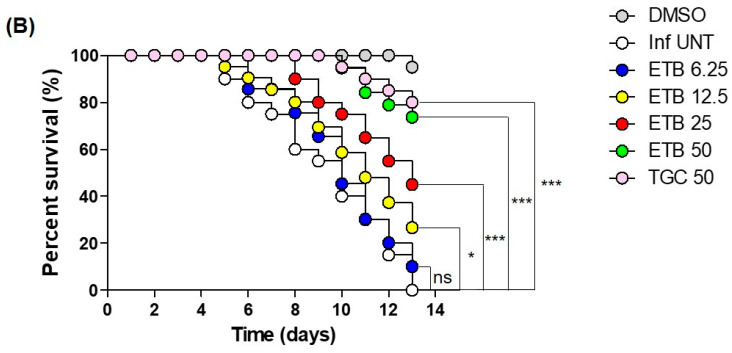
ZF In vivo efficacy of ETB. (**A**) Different concentrations of ETB (6.25, 12.5, 25, and 50 µM) and TGC (50 µM) were treated to the ZF infected with *Mab*R-mWasabi and proliferation or reduction of mWasabi signal in ZF was monitored under the fluorescent microscope. After antibiotics treatment, the bacterial burden of infected ZF was quantified through traditional agar plate quantification method. Data was expressed as the mean log10 CFU per embryo (*n* = 10 of each condition) from three independent experiments. (**B**) All infected fishes treated at 6.25, 12.5, 25, and 50 µM of ETB and TGC (50 µM). Survival curve was plotted from *Mab*R-mWasabi infected ZF for 13 days. (*n* = 20, representative of three independent experiments). Survival curves were compared with the log-rank (Mantel-Cox) test (* *p* < 0.05, ** *p* < 0.01; *** *p* < 0.001; ns: Not significant). Inf UNT: Infected but not treated control.

In vivo efficacy of ETB was also validated in *Danio rerio* (zebrafish; hereafter referred to as ZF) of infection. To determine whether ETB has the ability to treat *Mab* infected ZF as a therapeutic agent. ETB in vivo efficacy was evaluated in ZF after infection with *Mab* subsp. *abscessus* CIP104536^T^ R variant that express mWasabi green fluorescence (hereafter referred to as *Mab*R-mWasabi) at concentrations of 6.25, 12.5, 25, and 50 µM. First, we investigated whether the mWasabi fluorescent signal in infected ZF would be reduced by adding ETB in a dose-dependent manner. For this, dissemination of *Mab*R-mWasabi in ZF was monitored under a fluorescence microscope according to ETB concentrations. After the *Mab*R-mWasabi infection, ETB was administered at each different concentration (6.25, 12.5, 25, and 50 µM) for up to 5 days post-infection (dpi). As shown in Figure 4A, *Mab*R-mWasabi was disseminated and localized inside of ZF, especially in the head when the DMSO was treated. However, the mWasabi fluorescent signal was significantly reduced in the ETB treated condition. In more detail, a significant mWasabi reduction in the *Mab*R-mWasabi infected ZF head was observed at 25 μM ETB. Furthermore, almost no mWasabi protein signals were detected in the ZF when *Mab*R-mWasabi infected ZF were treated with 25 and 50 μM ETB. This mWasabi protein signal reduction at ETB 50 μM was similar to those of the positive control TGC treatment at 50 μM. To determine whether *Mab*R proliferated in the ZF body after treatment of ETB in different doses, each infected ZF was crushed, sampled and the bacterial burden was enumerated on a 7H10 Middlebrook agar plate. Figure 4A shows that *Mab*R could colonize the ZF and replicate inside the host and that the CFU showed significant differences between DMSO control and ETB treated groups. Until 6.25 μM of ETB treatment, there was no significant bacterial colony reduction on the 7H10 agar plate. However, after 12.5 μM of ETB treatment there started to be a reduction in the bacterial number. The smallest bacterial populations per ZF were found to occur in the presence of ETB 50 μM. This led to a 3.8 log_10_ reduction when compared to DMSO control.

Next, we evaluated the survival rate of *Mab*R infected ZF to know whether ETB could extend the lifespan of infected ZF after treatment of ETB dose-dependently. The survival rates of *Mab*R infected ZFs were observed for 13 days while the tank water was replaced using 6.25, 12.5, 25, and 50 μM of ETB daily. 50 μM of TGC treatment was used as a positive control and untreated ZF group was used as a negative control. ZF without *Mab*R infection was used as a mock control. The results shown in Figure 4B present the survival rates of the ETB-treated ZF cultures infected with *Mab*R compared with those of the untreated control ZF. 100% of fish that were infected by *Mab*R in the untreated group died at 13 days post infection (dpi). However, the ETB treated ZF group showed significantly extended lifespans. When *Mab*R infected ZF was exposed to 6.25 and 12.5 μM of ETB for 13 days, around 75 to 90% of the infected ZF died, similarly with the untreated group. However, when we increased the dosage of ETB to 25 and 50 μM, exponential extended lifespan was observed. The TGC (50 μM) treated ZF group also showed a similar significant lifespan-extending effect as was observed in the 50 μM ETB treatment group. Taken together, these results suggest that ETB has a therapeutic effect against *Mab* in vivo.

## 3. Discussion

In this study, we screened the Pandemic Response Box that consists of a set of 400 structurally diverse compounds. Using this chemical library, we have conducted dual screening with both *Mab* subsp. *abscessus* CIP 104536^T^ S- and R-variants. S-variants possess glycopeptidolipids (GPL) in the mycobacterial cell wall and minimal trehalose dimycolate (TDM) which contributes to the *Mycobacterium tuberculosis* virulence [18]. However, GPL is noticeably absent in R-variants and these R-variants are more virulent than S-variants in the animal model of infection by causing invasive infection. [20,21]. The R-variants could emerge from CF patients chronically colonized with an S strain, creating a more aggressive, invasive pulmonary infection. Thus, identification of a new compound that has more potency to the R-variant would be a beneficial to the patients who are suffering from R-variant infection. From the screen, we narrowed down 3 different hits named ETB, ERV, and BDQ that showed potent activity to both S and R-variants.

ERV is a synthetic halogenated tetracycline class and has a broad spectrum of activity against aerobic and anaerobic Gram-negative and Gram-positive bacteria [22]. ERV disrupts bacterial protein synthesis via binding to the 30S ribosomal subunit, thus preventing the incorporation of amino acid residues into elongating peptide chains [23]. In recent susceptibility tests against *Mab*, ERV showed 2–4 times lower in vitro MIC (0.125–2 mg/L) than tigecycline. Furthermore, ERV has improved intravenously administered pharmacokinetic/pharmacodynamic parameters and it is suggested that ERV could be more efficacious clinically than tigecycline [24].

BDQ was approved by the Food and Drug Administration and the European Medicines Agency for the treatment of multidrug-resistant tuberculosis (MDR-TB). It is a diarylquinoline antibiotic that targets the essential F_o_F_1_ ATP synthase [25,26]. BDQ has shown favorable in vivo activity against *Mab* infected animal models such as in ZF and the immunocompromised [27,28]. However, the efficacy of BDQ is somewhat doubtful and may depend on the animal model. For instance, BDQ did not decrease the bacterial burden after one month of treatment in a nude mouse that was athymic with a depletion of T cells [29]. In addition, Philley et al. reported preliminary effectiveness of BDQ-containing regimen treatment as salvage therapy for patients who have *Mab* lung disease. However, after 6 months of observation, only one patient among six showed an improvement of clinical symptoms [30]. Therefore, there is no clinical evidence that BDQ is a potential agent in the treatment of *Mab* infections.

In this study, we evaluated the activity of ETB in different infection models and it showed an excellent therapeutic effect against *Mab*. As shown in Table 1 and Table 2, survival of *Mab* was greatly reduced after ETB treatment in all the *Mab* strains tested in vitro, including *Mab* CIP 104536^T^ S- and R-variants and clinical isolates at a IC_50_ range of 0.29–2.75 µM. Furthermore, ETB could successfully inhibit spontaneously-induced drug-resistant *Mab* CIP 104536^T^ S strains that were generated previously [19]. In a time-kill assay, ETB exhibited dramatic concentration-dependent killing against *Mab* CIP 104536^T^ S at all tested drug concentrations (0.5–5× IC). Bacterial regrowth was not observed until 5 days after ETB treatment.

This in vitro activity was further validated using the mBMDMs model of infection. The intracellular activity of ETB against *Mab*, which resides in mBMDMs, was assessed using the Automated Cell Imaging System. The mBMDMs cells were infected with *Mab*S-mWasabi and the number of fluorescent mWasabi signals was statistically enumerated after treatment of ETB in a concentration-dependent manner. Using this system, we evaluated the intracellular activity and cytotoxicity of ETB in mBMDMs at the same time. As shown in Figure 3A, ETB showed significant fluorescent *Mab* reduction in a dose-dependent manner. ETB could completely inhibit fluorescence expressing *Mab* signals at 2.5 μM without cytotoxicity. IC_50_ of ETB against intracellular *Mab* was 1.7 µM (Figure 3B). This result showed comparable activity to TGC, which has bactericidal intracellular effect on *Mab* infecting THP-1 macrophages (Figure 4B) [31]. Thus, we demonstrated that ETB can successfully inhibit the growth of intracellular *Mab* without cytotoxicity.

The potent in vitro and intracellular inhibitory activity of ETB was further tested in the ZF infection model. ZF embryo caudal vein injection model is a popular tractable in vivo model that is often used to generate systemic infections for subsequent analyses, including survival experiments and bacterial burden determination [32]. This model has been successively used for in vivo anti-bacterial drug evaluations for *Mab* and *Mycobacterium* *marinum* [9,33,34,35,36,37]. For this reason, the ZF embryo infection model has been used as a preliminary in vivo efficacy evaluation before proceeding to a mouse in vivo efficacy evaluation in the drug discovery pipeline. Thus, we also evaluated the in vivo efficacy of ETB by injecting *Mab*R variant into ZF through caudal vein injection. In order to evaluate the toxicity of ETB, we evaluated the maximum tolerated dose (MTD) of ETB to ZF. Fifteen ZF were used in each treatment group. A broad range of ETB (ranging from 6.25 to 100 μM) was added to ZF-containing fish water, without bacterial infection. Around 100% of ZF died after 12 days of exposure to 100 μM ETB. Thus, less than 50 µM of ETB, which does not show significant lethality, were used for in vivo efficacy tests (data not shown). In vivo efficacy test, ETB treatment showed significant bacterial CFU reduction inside infected ZF in a dose-dependent manner in comparison with TGC treatment (Figure 4A). Furthermore, *Mab*R resulted in 100% of ZF death infected at 13 days post infection (dpi), while 50 µM ETB treatment resulted in a significantly enhanced lifespan. However, it is important to mention that ZF embryos model of infection also has some limitations. Firstly, adaptive immunity in ZF embryo is not fully matured until after 4–6 weeks [38,39]. Thus, it is likely to generate different outcomes than those obtained in mammalian models. Therefore, to show the effectiveness of the compound in a chronic model, it would be better to use immunocompromised mice which can allow sustained *Mab* pulmonary infection [40]. Second, some compounds have a capacity to persistently adhere to the ZF skin during the treatment procedure resulting in an artificial carryover effect in the evaluation of in vivo efficacy through bacterial CFU quantification [36]. Moreover, pharmacokinetics are not known in ZF which makes it difficult to directly transpose the treatment dose obtained from ZF to humans [35]. Thus, a drug efficacy evaluating system using ZF-*Mab* infection should be regarded as an early model for screening new bio-active compounds using the chemical library or preclinical drug testing. These should then be evaluated in other higher animal models before clinical trials.

ETB is a benzoxaboroles analogue and leucyl-tRNA synthetase (LeuRS) inhibitor and it has a novel mode of action against Gram-negative bacterial infection such as urinary tract infections [41,42]. The LeuRS catalyzes the ATP-dependent ligation of L-leucine to tRNA. In previous studies, the benzoxaborole analogue (referred as compound 1) also showed antitubercular activity against *M. tuberculosis* (MIC of 0.26 µg/mL) and *M. smegmatis* (MIC of 1 µg/mL) [43]. Furthermore, a spontaneous mutant was generated from both species on a Middlebrook 7H10 agar plate containing benzoxaborole analogue at 5× or 10× MIC_99_ and spontaneous mutants were shown to be harboring an amino acid substitution on mycobacterial LeuRS. Intriguingly, *Mab* also contains *leuS* (MAB_4923c) which has a 75% and 78% amino acid homology with *M. tuberculosis* and *M. smegmatis* respectively. Thus, ETB might share the same mechanism of action with the benzoxaborole analogue (compound 1) which has demonstrated its activity against *M. tuberculosis* and *M. smegmatis*. Based on ClinicalTrials.gov in 2017, a clinical phase II study of ETB for the treatment of complicated urinary tract infection and complicated intra-abdominal infection was terminated due to the rapid emergence of drug resistance during treatment [41]. Recently however, a new oxaborole inhibitor, DS86760016, has reignited the re-use of the LeuRS inhibitor resulting in a lower frequency of resistance development than ETB in comparative murine urinary tract infection models. Unfortunately, DS86760016 is not commercially available currently because Daiichi Sankyo India, the pharmaceutical company that discovered DS86760016, was closed in 2017 (personal communication with Dr. Nobuhisa Masuda). For this reason, we synthesized DS86760016 through a collaboration with the medicinal chemistry team based on the structure-activity relationship. Currently, we have checked its activity for *Mab* and it showed similar IC value with ETB against *Mab* (data not shown). The newly synthesized DS86760016 will be further tested in several models of infection to know its anti-*Mab* activity and resistant mutant frequency in comparison with ETB soon.

In this study, we screened the Pandemic Response Box and identified several hits in the concentration-response curve. Among these hits, ETB showed excellent in vitro activity against 3 different *Mab* subspecies, various clinical isolates, and drug resistant strains. In addition, ETB showed significant intracellular *Mab* killing activity at low concentrations. It also showed a terrific therapeutic effect in the *Mab* infected ZF models. Therefore, ETB is a potential anti-*Mab* drug candidate that can be further developed for the anti-*Mab* drug discovery pipeline.

## 4. Materials and Methods

### 4.1. Bacterial Strains and Culture Conditions

*Mab* subsp. *abscessus* CIP 104536^T^ S- and R-variants were kindly provided by Dr. Laurent Kremer (CNRS, IRIM, Universite’ de Montpellier, Montpellier, France). *Mab* subsp. *bolletii* CIP108541^T^ and *Mab* subsp. *massiliense* CIP108297^T^ were obtained from the Collection de l’Institut Pasteur (CIP, Paris, France). Clinical isolates were purchased from the Korea Mycobacterium Resource Center (KMRC, Osong, Korea). *Mab* strains were grown in Middlebrook 7H9 broth (BD Biosciences, San Hose, CA, USA) supplemented with 10% (*v*:*v*) albumin, dextrose, and catalase (ADC; BD), 0.2% (*v*:*v*) glycerol and 0.05% (*v*:*v*) Tween 80 or Middlebrook 7H10 agar plates (BD) containing 10% (*v*:*v*) oleic acid and ADC (OADC; BD) enrichment and 0.5% (*v*:*v*) glycerol. All cultures were grown at 37 °C with shaking at 180 rpm. CLA and ETB were purchased from Sigma-Aldrich (St. Louis, MO, USA) and MedKoo Biosciences, Inc. (Cat#: 319569; Morrisville, NC, USA) respectively. TGC was purchased from Adooq Bioscience (Cat#: A10933; Irvine, CA, USA). For the mBMDM preparation and ZF infection, recombinant *Mab* CIP 104536^T^ S and R that carrying a pMV262-mWasabi plasmid expressing mWasabi protein was prepared as previously described [33].

### 4.2. Resazurin Microtiter Assay (REMA)

Fifty microliters of *Mab* culture (approximately 5 × 10^4^ CFU/mL) in Middlebrook 7H9 broth supplemented with 10% ADC, 0.2% glycerol was used per well and 50 μL of serial 2-fold dilutions of test compound solution was added to each well of a sterile, polystyrene 96-well cell culture plate (SPL; Gyeonggi-do, Korea). A drug-free growth control was included in each plate. 200 μL of sterile water was added to outer perimeter wells to prevent evaporation during incubation. Plates were then covered with self-adhesive membranes and incubated at 37 °C for 3 days. The resazurin solution was prepared as a 0.025% (*w*/*v*) solution in sterile distilled water using resazurin sodium salt powder (Sigma, St. Louis, MO, USA), filter sterilized. After 3 days of incubation, 40 μL of the resazurin solution was added to the wells. Fluorescence was measured using a SpectraMax^®^ M3 Multi-Mode Microplate Reader (Molecular Devices, Sunnyvale, CA, USA). Dose response curve was constructed and concentrations required to inhibit bacterial growth by 50% (IC50) was determined the GraphPad Prism software (version 6.05; San Diego, CA, USA).

### 4.3. ETB Time-Kill Assay for Mab

An early exponential phase mycobacterial culture (10^7^ CFU/mL) was prepared in 30 mL of 7H9 broth supplemented with 10% ADC, 0.2% glycerol. Two-fold increasing concentrations of ETB (from 0.5 to 5× MIC), 5× MIC of CLA and a drug-free growth control was used. To perform CFU counting, 100 μL samples were taken from each bottle, and 10-fold dilutions of samples were then made in PBS (900 μL PBS, 100 μL sample) at different time intervals (0, 1, 2, 3, 4, and 5 days). A total of 50 μL of each dilution was plated onto 7H10 agar, containing 10% OADC supplement and bactericidal activity of ETB was compared to the drug-free growth control. All time-kill experiments were performed in triplicate and the mean CFU counts plotted.

### 4.4. Intracellular Bacterial Replication Assays

Intracellular activity of ETB was analyzed as previously described [44]. Briefly, differentiated mBMDMs (7 × 10^5^ cells/well) cells were infected with *Mab*S-mWasabi at a multiplicity of infection (MOI) of 1:1. After 3 h of infection, the cells were washed with PBS (phosphate-buffered saline) and incubated with DMEM (Dulbecco’s Modified Eagle’s medium) containing 250 μg/mL amikacin for 1 h to kill the extracellular *Mab*S-mWasabi [45]. The cells were washed with PBS and then dispensed into 96-well plates (Corning, New York, NY, USA). The broth medium, with a 2-fold serial dilution of compound, was subjected to a 96-well plate. For all experiments, the amount of DMSO was maintained at a 1% final concentration per well. After 3 days of incubation at 37 °C and 5% CO_2_, macrophages were stained for 1 h with 5 μM Syto 60 dye (Thermo Fisher Scientific, Waltham, MA, USA), and images were recorded using the ImageXpress Pico Automated Cell Imaging System (Molecular Devices, Sunnyvale, CA, USA). The number of bacteria in macrophage and macrophage number were quantified using the CellReporterXpress^®^ Image Acquisition and Analysis Software (Molecular Devices).

### 4.5. Drug Efficacy Assessment in MabR-mWasabi Infected ZF

ZF experiments were approved by the Animal Research Ethics Committee of Gyeongsang National University (Project identification code: GNU-190325-E0014, Approval date: 25 March 2019). ZF larvae at 30–48 h post-fertilization were dechorionated and tricaine (270 mg/L) was used for ZF larvae anesthesia. Mid-log-phase cultures of *Mab*R-mWasabi were used for ZF larvae infection as described previously [35]. Around 3 nL of *Mab*R-mWasabi (400 CFU) was injected via caudal vein using a Tritech Research Digital microINJECTOR^TM^, (MINJ-D; Tritech research, CA, USA). The accurate infected CFU was enumerated by CFU quantification method using 7H10 agar plates containing 10% OADC enrichment and 0.5% glycerol with kanamycin (50 mg/L). The infected larvae were transferred into 96-well plates (2 fishes/well) and incubated at 28.5 °C after treatment of compound. Different concentration of ETB (ranges from 6.25 to 50 µM at the final concentration) and TGC (50 µM) were added directly into the blue fish water (methylene blue 300 µL/L). The fish water and compound were renewed once daily and each different concentration of compound was absorbed orally by infected larvae. The embryos survival was monitored and dead embryos (no heartbeat) were recorded on daily to determine survival curve. The DMSO was used as negative. The in vivo efficacy of ETB was evaluated by quantification of *MabR* load in ZF by counting serial dilutions of bacterial CFU as previous described [9,33,44] The CFU quantification and survival curve were plotted by GraphPad Prism software using the method from Kaplan and Meier, and log-rank (Mantel–Cox) test respectively.

## Figures and Tables

**Table 1:** Chemical structure and half maximal inhibitory concentration (IC_50_) values of the 3 most potent *Mab* hits.

	Compound ID	Disease Set	Trivial Name	IC50 (µM)	Structure
Pandemic Response Box Hits (<20 μM)	MMV1578566	Anti-bacterial	Epetraborole (ETB)	0.4	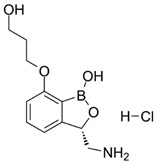
	MMV1578574	Anti-bacterial	Eravacycline (ERV)	0.1	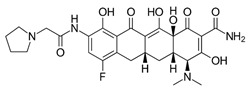
	MMV689758	Anti-bacterial	Bedaquiline (BDQ)	0.8	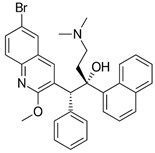
			Clarithromycin (CLA)	1.6	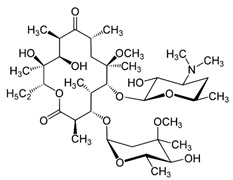

**Table 2:** Inhibitory potency of ETB against *Mab* subspecies, clinical isolates, and drug resistant *Mab* strains.

*Mab* Subsp.	Colony Morphology	Cation-Adjusted Mueller-Hinton (CAMH) Medium	
		IC_50_ (μM)	IC_90_ (μM)
*abscessus* CIP104536	R	0.25	0.56
*bolletii* CIP108541	S	0.22	0.56
*massiliense* CIP108297	S	0.11	0.33
*abscessus* KMRC 00136-61038	S	1.24	2.47
*abscessus* KMRC 00136-61039	S	0.29	1.21
*abscessus* KMRC 00136-61040	R	2.57	10.36
*abscessus* KMRC 00136-61041	S	0.74	1.57
*abscessus* KMRC 00200-61199	S	1.47	4.31
*abscessus* KMRC 00200-61200	S	0.64	2.78
*abscessus* KMRC 00200-61201	S	0.60	1.34
*massiliense* KMRC 00200-61202	R	2.75	4.94
*massiliense* KMRC 00200-61204	S	1.41	2.74
*M. abscessus* (CLA-R)	S	0.31	1.17
*M*. *abscessus* (AMK-R)	S	0.35	1.16
*M*. *abscessus* (CFX-R)	S	0.35	0.93
